# Supplementary material for: Differentiation of adsorption and degradation in steroid hormone micropollutants removal using electrochemical carbon nanotube membrane
Source: Nat Commun. 2024 Nov 4;15:9524. doi: 10.1038/s41467-024-52730-7 (PMC11535516; doi:10.1038/s41467-024-52730-7)
Supplement: Supplementary file 1 — Supplementary Information [file 41467_2024_52730_MOESM1_ESM.pdf]

Supplementary Information

Differentiation of adsorption and degradation in steroid hormone micropollutants removal using electrochemical carbon nanotube membrane

Siqi Liu[1], David Jassby[2], Daniel Mandler[3], & Andrea I. Schäfer[1]*

[1]*Institute for Advanced Membrane Technology (IAMT), Karlsruhe Institute of Technology (KIT), Eggenstein-Leopoldshafen, Germany*

[2]*Department of Civil and Environmental Engineering, University of California, Los Angeles, Los Angeles, United States*

[3]*Institute of Chemistry, The Hebrew University of Jerusalem, Jerusalem, Israel.*

*Corresponding author: Andrea.Iris.Schaefer@kit.edu (A. I. Schäfer), +49 (0)721 6082 6906

Revision submitted to
Nature Communications
Special Issue: Water Treatment and Harvesting
29/08/2024

Supplementary Discussion 1. Contribution of adsorption and degradation to electrochemical removal of estradiol

To study the electrochemical removal process as a function of the filtration volume, the mass balance analysis was conducted for the experiment at standard conditions within different permeate volume range (Supplementary Figure 1).

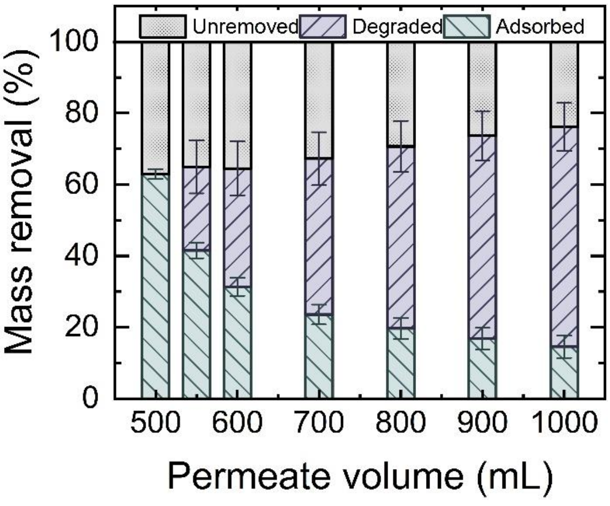

Supplementary Figure 1. Contribution of electrochemical adsorption and degradation to the mass removal of E2 within the CNT EMR as a function of the permeate volume. $c_{f,E2}$ = 1 µg L^{-1}, V_{cell} = 1.6 V, J_f = 150 L m^{-2} h^{-1} (5 mL min^{-1}), 1 mM $NaHCO_3$, 10 mM NaCl, 27.2 mg L^{-1} EtOH, 79.2 mg L^{-1} MeOH, pH 8.2±0.2, 23±1 ℃. Voltage on at 500 mL. Error bars represent propagated error from operational parameter variations and analytical error.

At 500 mL of permeate, the total mass of removed E2 was 63±1%, solely attributed to adsorption. Upon activating the voltage at 500 mL, total mass removal increased from 63±1% to 76±10% as the permeate volume increased from 500 to 1000 mL.

The contribution from degradation to total mass removal grew from 0 to 61±7% over the same volume range.

Meanwhile, the contribution from adsorption decreased from 63±1% to 19±3% as the permeate volume increased from 500 to 800 mL and then stabilized upon further increasing the volume to 1000 mL.

Supplementary Discussion 2. Electrochemical degradation of steroid hormone at varying parameters

The influence of cell voltage, water flux, initial concentration and SH type on the electrochemical degradation was investigated. The profiles of the normalized SHs concentration and the evolution of UHPLC-FSA chromatograms *vs.* cumulative permeate volume for all the electrochemical experiments are shown below. To ascertain the stable conditions during the electrochemical degradation experiments, the variations in the parameters, including pH, transmembrane pressure, conductivity, and temperature were monitored and recorded along with the experiments.

Data for experiments at varying cell voltage

Supplementary Figure 2 and Supplementary Figure 3 show the changes of the normalized SHs concentration and the evolution of UHPLC-FSA over 1 L of cumulative permeate volume for experiments at varying cell votage. Supplementary Figure 4 presents the variation of the system conditions during the experiments.

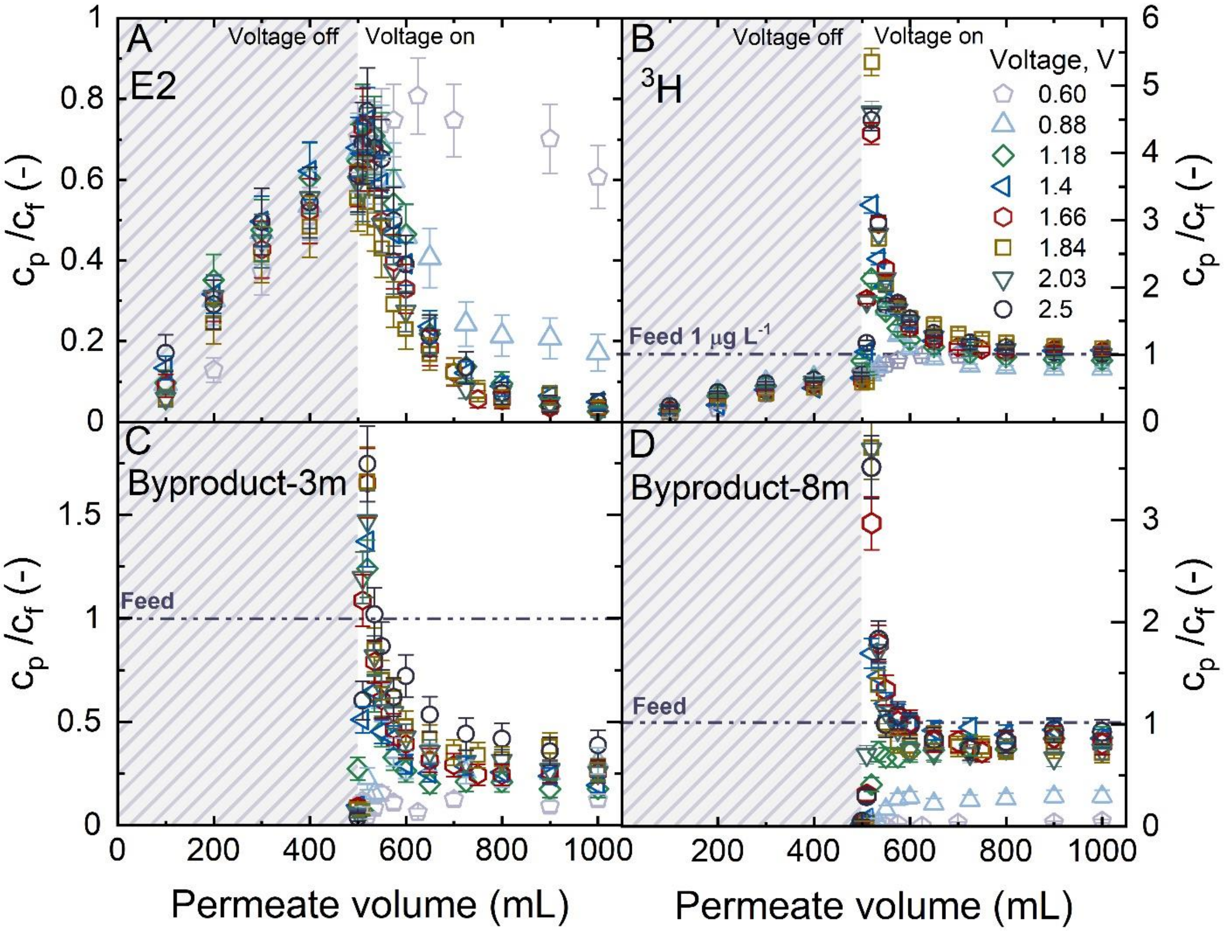

Supplementary Figure 2. Effect of cell voltage on the electrochemical degradation of E2 expressed as normalized (A) E2 concentration in permeate (c_p/c_f), (B) ^{3}H activity in permeate ($c_{p,3H}/c_{f,3H}$), (C) concentration of byproduct-3m ($c_{p,\mathrm{prod3}}/c_{f,\mathrm{prod3}}$), and (D) concentration of byproduct-8m ($c_{p,\mathrm{prod8}}/c_{f,prod8}$) in the permeate *vs.* accumulated permeate. $c_{f,E2}$ = 1 μg L^{-1}, J_f = 150 L m^{-2} h^{-1} (5 mL min^{-1}), 1 mM $NaHCO_3$, 10 mM NaCl, 27.2 mg L^{-1} EtOH, 79.2 mg L^{-1} MeOH, pH 8.2±0.2, 23±1 °C. Error bars represent propagated error from operational parameter variations and analytical error.

The steady-state normalized concentration of E2 in the permeate significantly decreased from 0.61±0.07 to 0.03±0.01 when the cell voltage was raised from 0.6 to 1.2 V, thereafter stabilizing upon further increasing the voltage to 2.5 V.

Beyond a cell voltage of 1.2 V, the normalized concentration of ^{3}H surpassed 1 immediately after the voltage was applied and continued to rise steadily with additional voltage increases. Following a cumulative permeate volume of 700 mL, the normalized concentration of ^{3}H leveled off, approaching

1. Concurrently, the normalized concentrations of the two byproducts in the permeate exhibited an increase in tandem with the cell voltage.

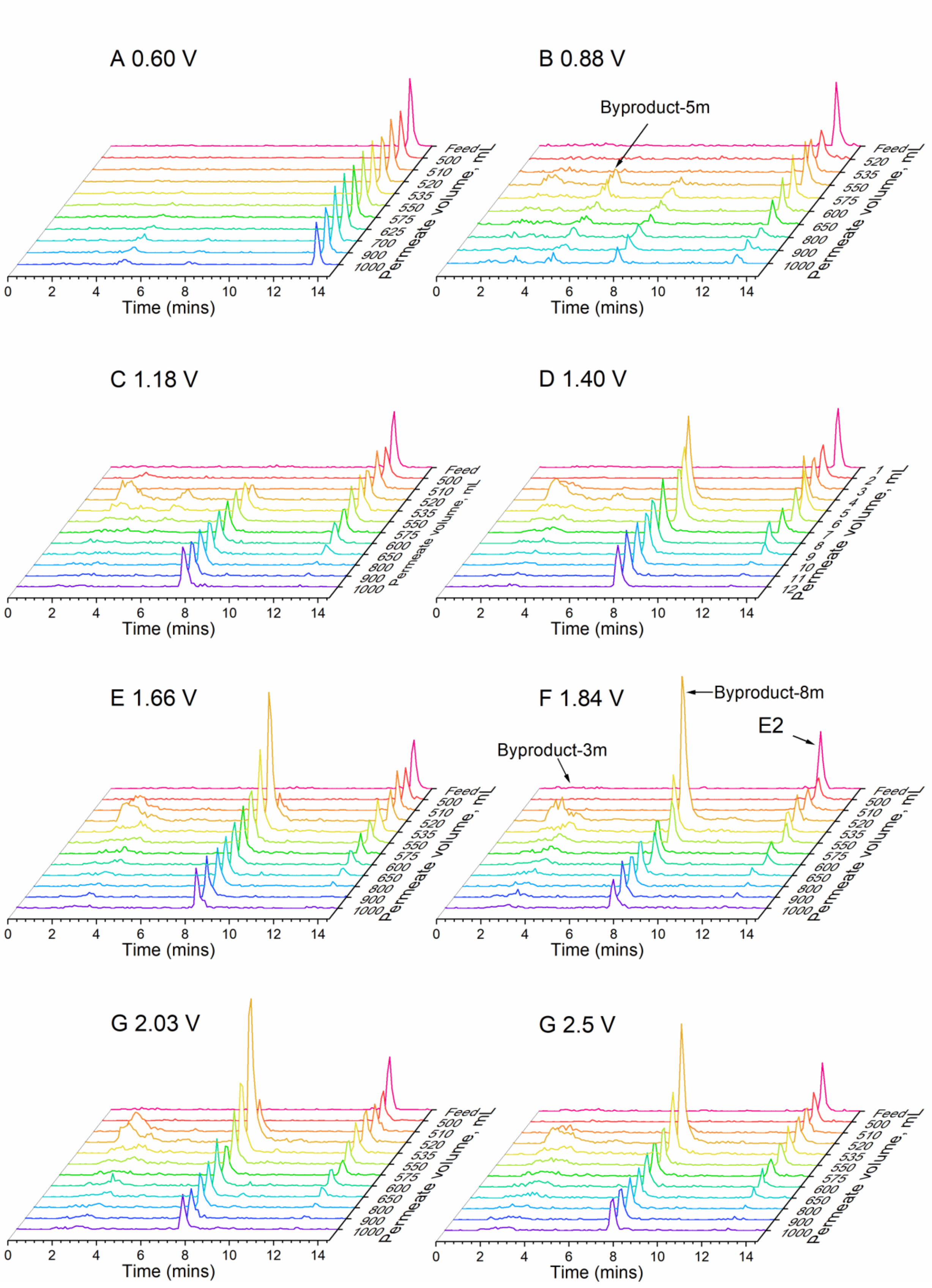

Supplementary Figure 3. UHPLC-FSA chromatograms of E2 during the electrochemical degradation with increasing accumulated permeate volume at varying cell voltage (A) 0.88, (B)

1.18, (C) 1.4, (D) 1.66, (E) 1.84, (F) 2.03, and (G) 2.5 V. $c_{f,E2}$ = 1 µg L^{-1}, J_f = 150 L m^{-2} h^{-1} (5 mL min^{-1}), 1 mM $NaHCO_3$, 10 mM NaCl, 27.2 mg L^{-1} EtOH, 79.2 mg L^{-1} MeOH, pH 8.2±0.2, 23±1 °C.

No discernible peaks corresponding to byproducts were detected at 0.6 V, whereas at 0.9 V, three byproduct peaks appearing at 3, 5, and 8 minutes. With an increase in voltage, the peak associated with byproduct-5m vanished.

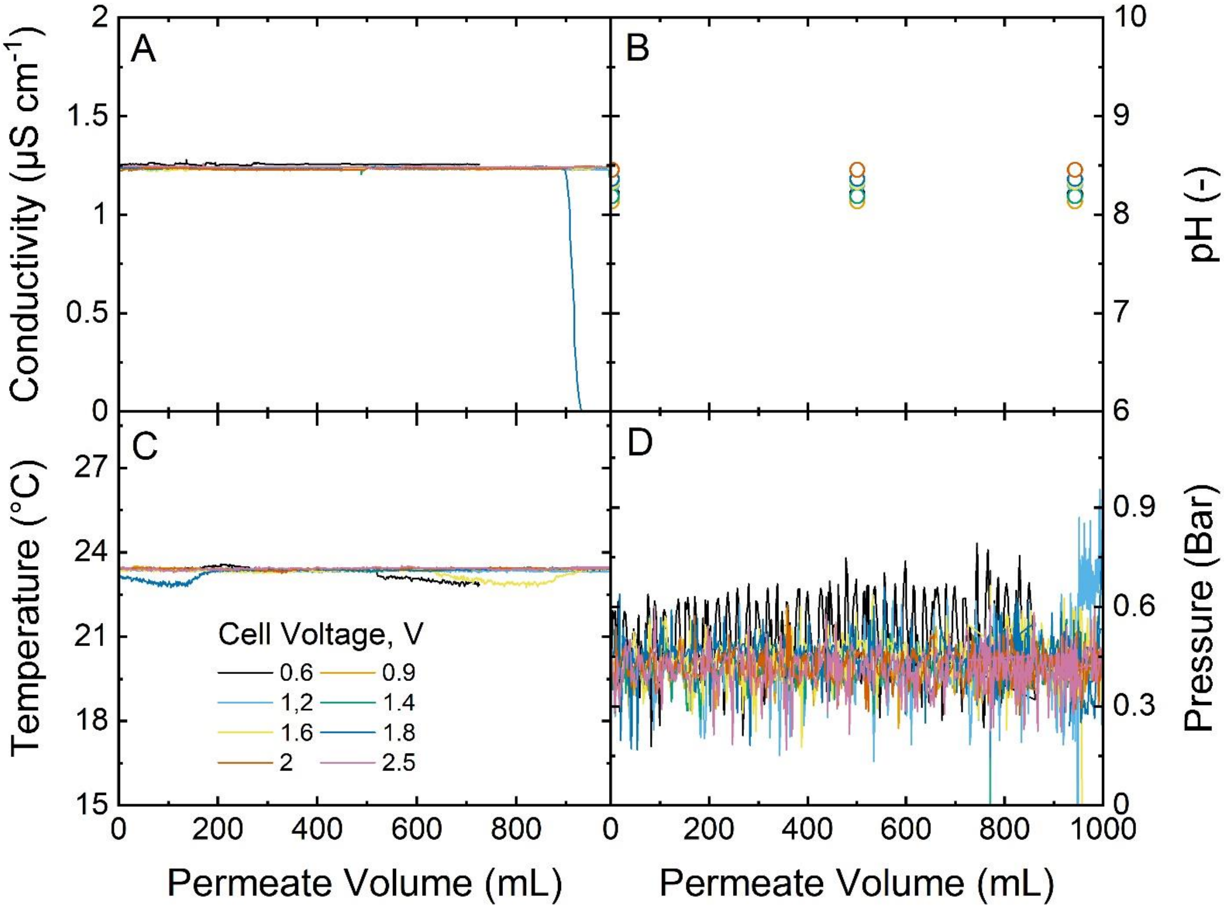

Supplementary Figure 4. Variations of (A) conductivity, (B) pH, (C) temperature conductivity, and (D) transmembrane pressure during electrochemical filtration experiments at varying cell voltage. $c_{f,E2}$ = 1 µg L^{-1}, J_f = 150 L m^{-2} h^{-1} (5 mL min^{-1}), 1 mM $NaHCO_3$, 10 mM NaCl, 27.2 mg L^{-1} EtOH, 79.2 mg L^{-1} MeOH, pH 8.2±0.2, 23±1 °C.

The system parameters remained stable during the filtration exeperiments.

Data for experiments at varying flux

Supplementary Figure 5 and Supplementary Figure 6 show the changes of the normalized SHs concentration and the evolution of UHPLC-FSA over 1 L of cumulative permeate volume for experiments at varying flux. Supplementary Figure 7. presents the variation of the system conditions during the experiments.

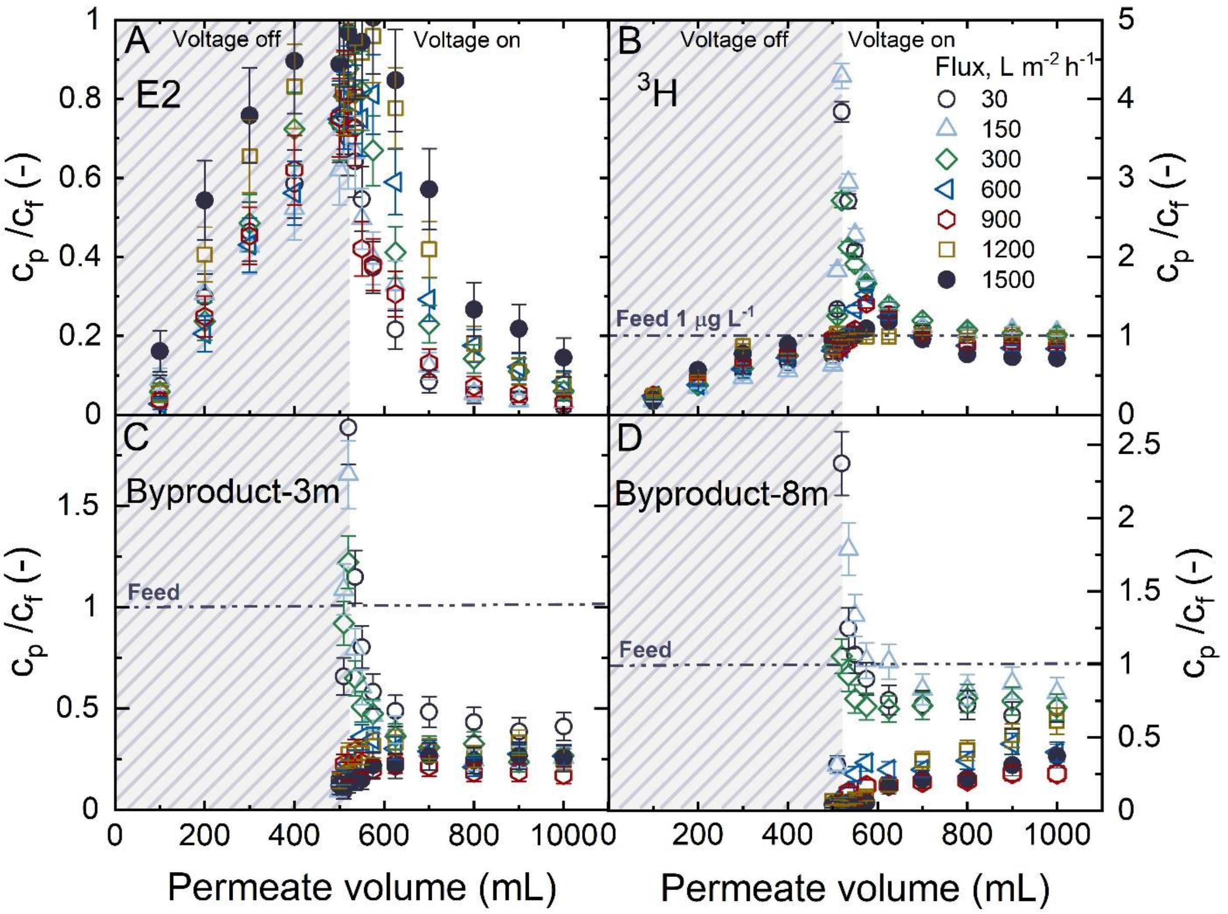

Supplementary Figure 5. Effect of water flux on the electrochemical degradation of E2 expressed as normalized (A) E2 concentration in permeate ($c_{p,E2}/c_{f,E2}$), (B) ^{3}H activity in permeate ($c_{p,3H}/c_{f,3H}$), (C) concentration of byproduct-3m ($c_{p,\mathrm{met3}}/c_{f,met3}$), and (D) concentration of byproduct-8m ($c_{p,\mathrm{met8}}/c_{f,met8}$) in the permeate *vs.* accumulated permeate. $c_{f,E2}$ = 1 µg L^{-1}, V_{cell} = 1.66 V, 1 mM $NaHCO_3$, 10 mM NaCl, 27.2 mg L^{-1} EtOH, 79.2 mg L^{-1} MeOH, pH 8.2±0.2, 23±1 °C. Error bars represent propagated error from operational parameter variations and analytical error.

Elevating the flux from 30 to 1500 L m^{-2} h^{-1} had a negligible impact on the steady-state concentration of E2, which observed a slight increase from 0.02±0.01 to 0.14±0.05.

After activating the voltage, the normalized concentration of ^{3}H in the permeate rose as the flux decreased. Similarly, the normalized concentration of byproducts in the permeate also augmented with the reduction in flux.

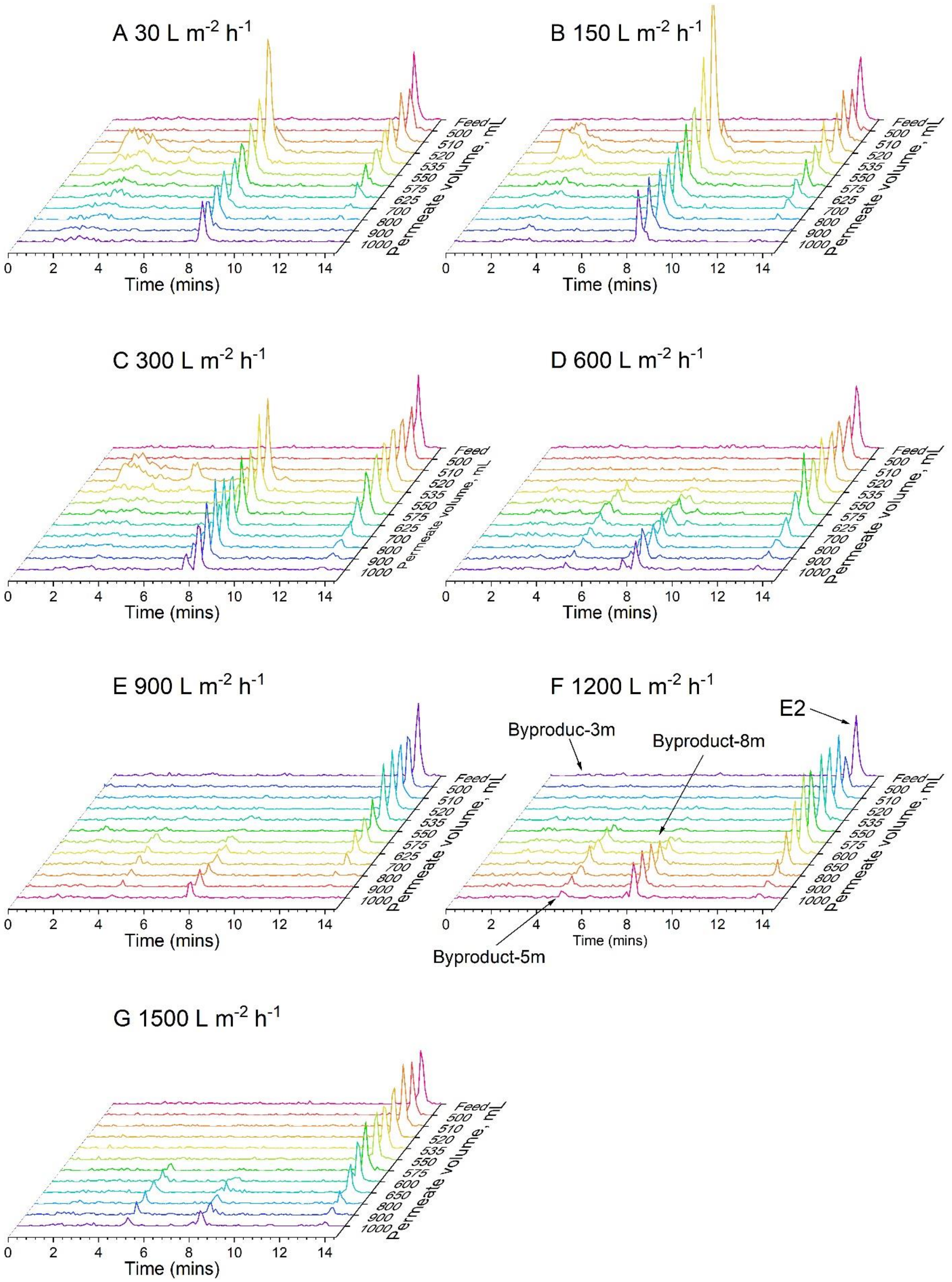

Supplementary Figure 6. UHPLC-FSA chromatograms of E2 during the electrochemical degradation with increasing accumulated permeate volume at varying flux (A) 30, (B) 150, (C) 600, (D) 900, (E) 1200 and (F) 1500 L m^{-2} h^{-1}. $c_{f,E2}$ = 1 µg L^{-1}, V_{cell} = 1.6 V, 1 mM $NaHCO_3$, 10 mM NaCl, 27.2 mg L^{-1} EtOH, 79.2 mg L^{-1} MeOH, pH 8.2±0.2, 23±1 °C.

In the UHPLC chromatograms, two byproducts appearing at approximately 3 and 8 minutes were detected at flux of 30 and 150 L m^{-2} h^{-1}.

A byproduct emerging at 5 minutes became evident when the flux was increased to 300 L m^{-2} h^{-1}. The peak area of byproduct-5m initially rose with an increase in flux to 600 L m^{-2} h^{-1} and then declined upon further increasing the flux to 1500 L m^{-2} h^{-1}.

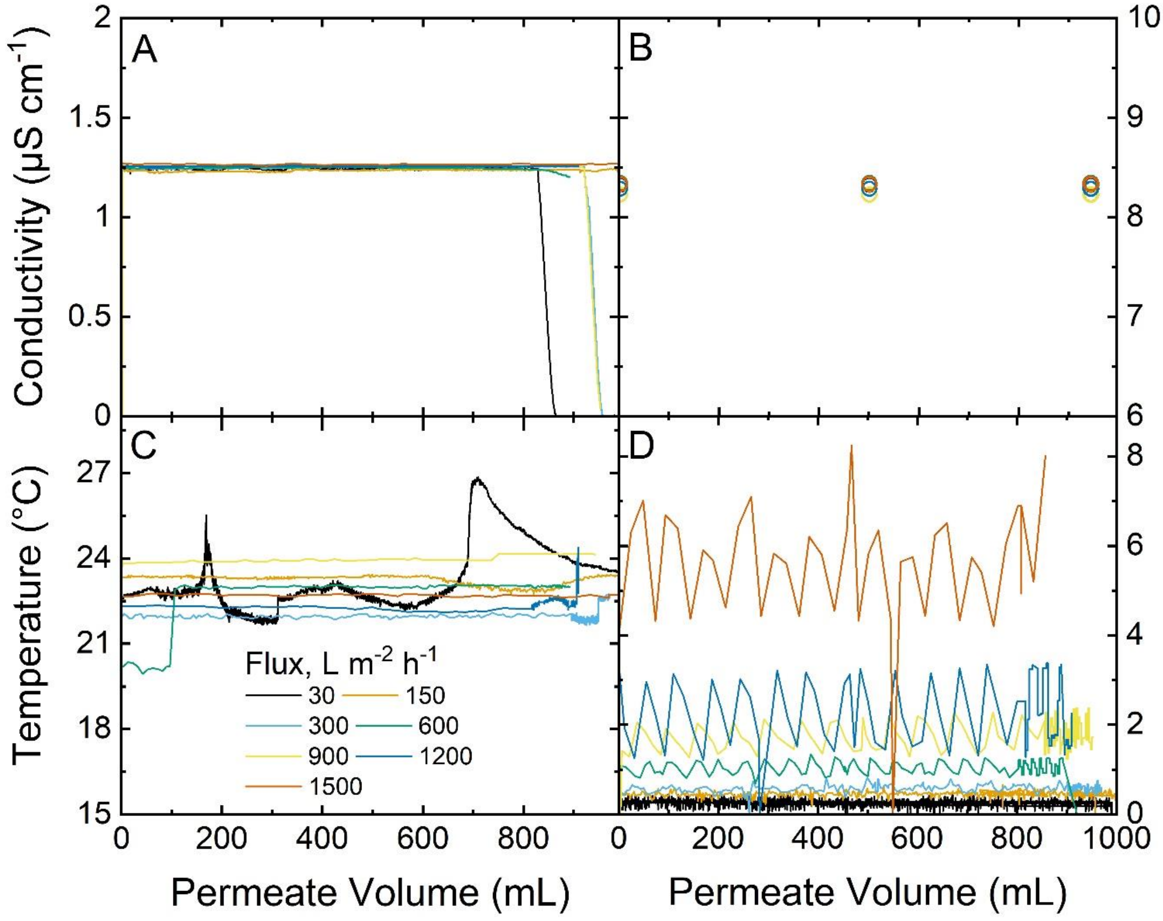

Supplementary Figure 7. Variations of (A) conductivity, (B) pH, (C) temperature conductivity, and (D) transmembrane pressure during electrochemical filtration experiments at varying flux. $c_{f,E2}$ = 1 µg L^{-1}, V_{cell} = 1.6 V, 1 mM $NaHCO_3$, 10 mM NaCl, 27.2 mg L^{-1} EtOH, 79.2 mg L^{-1} MeOH, pH 8.2±0.2, 23±1 °C.

The temperature at 30 L m^{-2} h^{-1} exhibited large variation, which was likely due to the sensors being accidentally touched by the operator.

The large variation in pressure at high flux may be attributed to the pulsating nature of the peristaltic pump. Peristaltic pumps operate by compressing and releasing a length of tubing, inherently producing a pulsating flow. This effect tends to be more significant at higher fluxes, where the pulsations can greatly influence pressure stability.

Data for experiments at varying steroid hormone concentration

Supplementary Figure 8. and Supplementary Figure 9. show the changes of the normalized SHs concentration and the evolution of UHPLC-FSA over 1 L of cumulative permeate volume for experiments at varying E2 concentration. Supplementary Figure 10. presents the variation of the system conditions during the experiments.

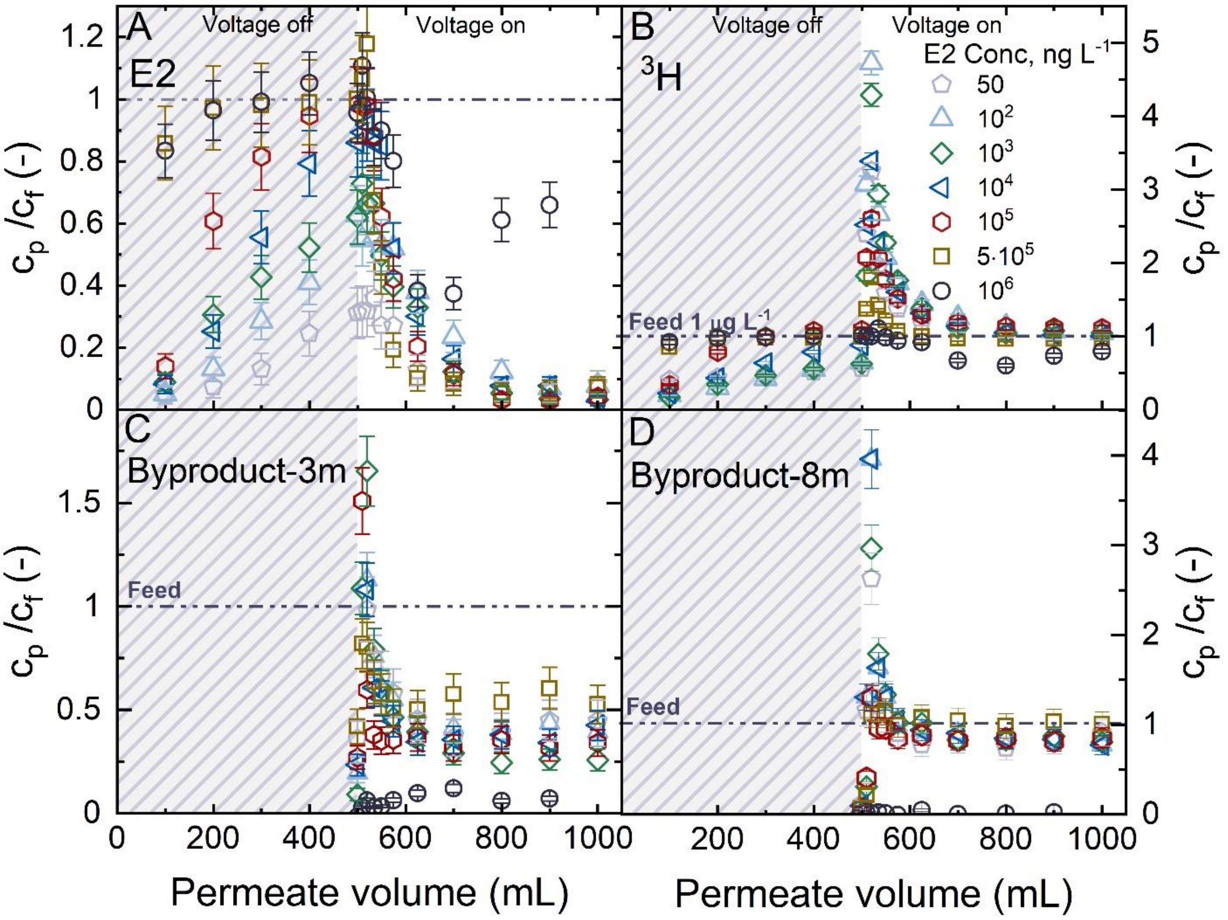

Supplementary Figure 8. Effect of SH concentration on the electrochemical degradation of E2 expressed as normalized (A) E2 concentration in permeate ($c_{p,E2}/c_{f,E2}$), (B) ^{3}H activity in permeate ($c_{p,3H}/c_{f,3H}$), (C) concentration of byproduct-3m ($c_{p,\mathrm{met3}}/c_{f,met3}$), and (D) concentration of byproduct-8m ($c_{p,\mathrm{met8}}/c_{f,met8}$) in the permeate *vs.* accumulated permeate. V_{cell} = 1.6 V, J_f = 150 L m^{-2} h^{-1} (5 mL min^{-1}), 1 mM $NaHCO_3$, 10 mM NaCl, 27.2 mg L^{-1} EtOH, 79.2 mg L^{-1} MeOH, pH 8.2±0.2, 23±1 °C. Error bars represent propagated error from operational parameter variations and analytical error.

At elevated concentrations of E2, the membrane becomes saturated with E2 more quickly.

The steady-state normalized concentration of E2 remained relatively constant across the concentration range of 50 to 500,000 ng L^{-1}, but sharply increased to 0.66±0.07 at an E2 concentration of 10^6 ng L^{-1}.

After activating the voltage, the normalized concentration of ^{3}H in the permeate decreased with an increase in E2 concentration from 10^2 to 10^6 ng L^{-1}, a trend that was similarly observed for the normalized concentration of byproducts in the permeate.

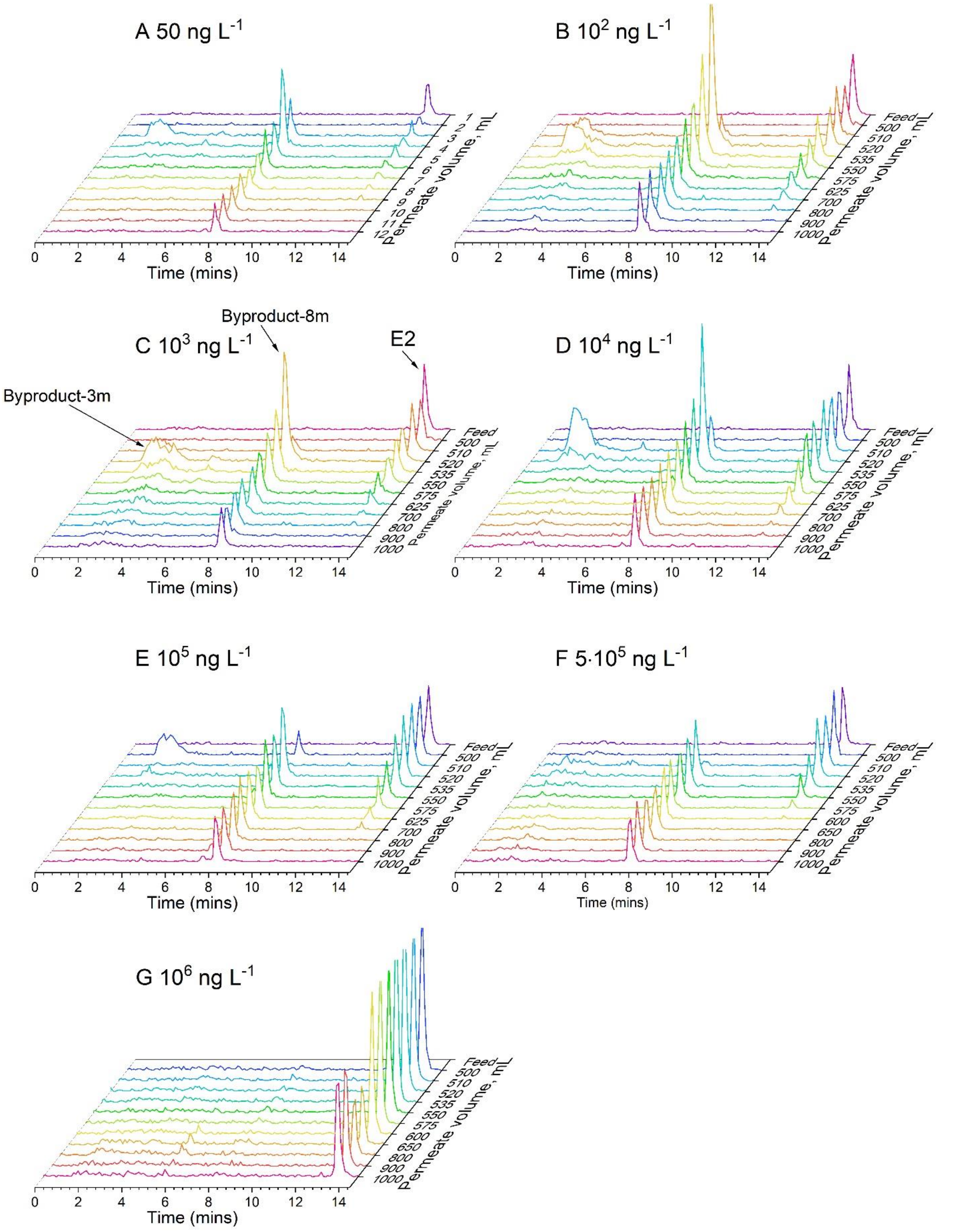

Supplementary Figure 9. UHPLC-FSA chromatograms of E2 during the electrochemical degradation with increasing accumulated permeate volume at varying flux (A) 50, (B) 10^2, (C) 10^3,

(D) 10^4, (E) 10^5, (F) $5{\cdot}10^5$, and (G) 10^6 ng L^{-1}. $c_{f,E2}$ = 1 µg L^{-1}, V_{cell} = 1.6 V, J_f = 150 L m^{-2} h^{-1} (5 mL min^{-1}), 1 mM $NaHCO_3$, 10 mM NaCl, 27.2 mg L^{-1} EtOH, 79.2 mg L^{-1} MeOH, pH 8.2±0.2, 23±1 °C.

The peak area of the byproducts showed a consistent decrease as the E2 concentration increased from 10^2 to 10^6 ng L^{-1}. However, this decrease should not be interpreted as fewer byproducts being generated at higher concentrations.

The reason is that feed solutions with E2 concentrations above 100 ng L^{-1} were composed of a mixture of radiolabeled and non-labeled E2, and the non-labeled E2 was not detectable using the UHPLC-FSA method.

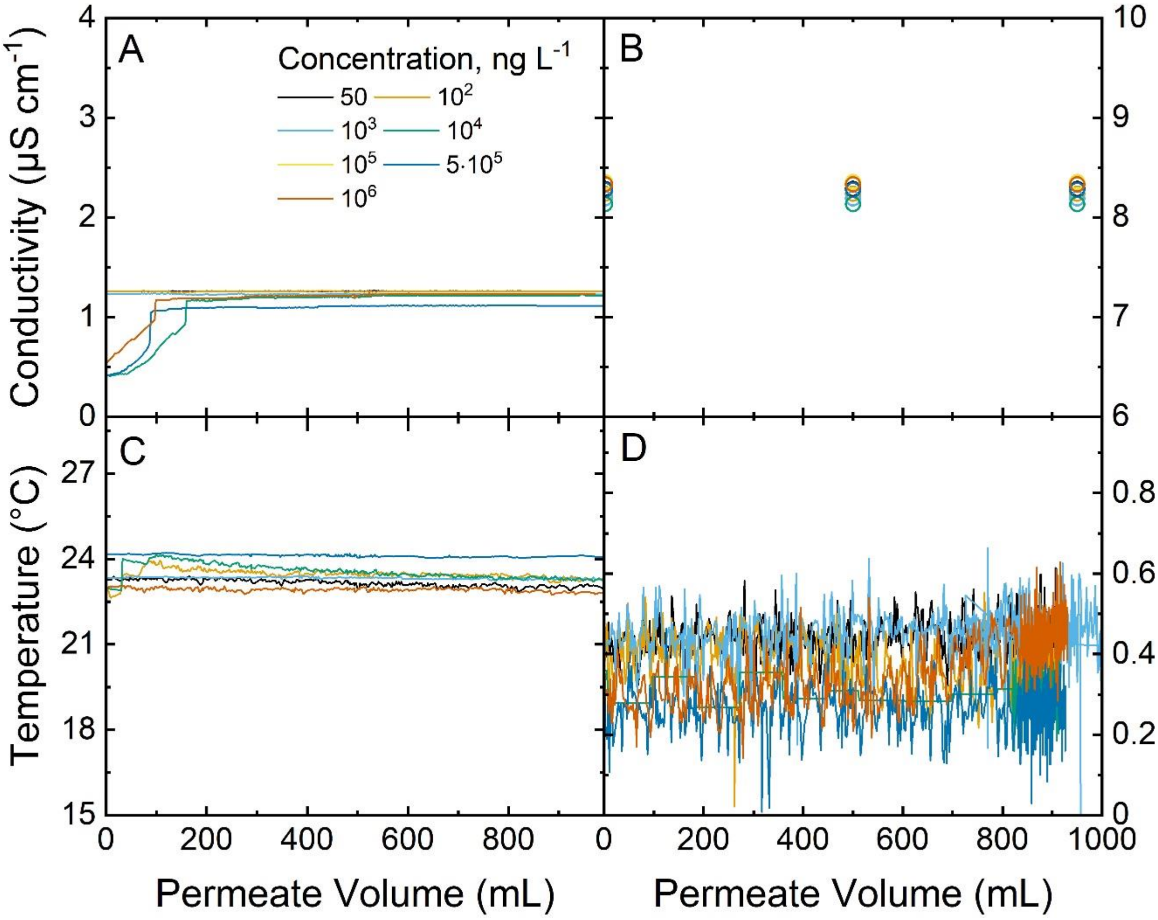

Supplementary Figure 10. Variations of (A) conductivity, (B) pH, (C) temperature conductivity, and (D) transmembrane pressure during electrochemical filtration experiments at varying concentration. $c_{f,E2}$ = 1 µg L^{-1}, V_{cell} = 1.6 V, J_f = 150 L m^{-2} h^{-1} (5 mL min^{-1}), 1 mM $NaHCO_3$, 10 mM NaCl, 27.2 mg L^{-1} EtOH, 79.2 mg L^{-1} MeOH, pH 8.2±0.2, 23±1 °C.

The system parameters remained stable during the filtration exeperiments.

Data for experiments at varying steroid hormone types

Supplementary Figure 11. shows the changes of the normalized SHs concentration and the evolution of UHPLC-FSA over 1 L of cumulative permeate volume for experiments at varying SH types. Supplementary Figure 12. presents the variation of the system conditions during the experiments.

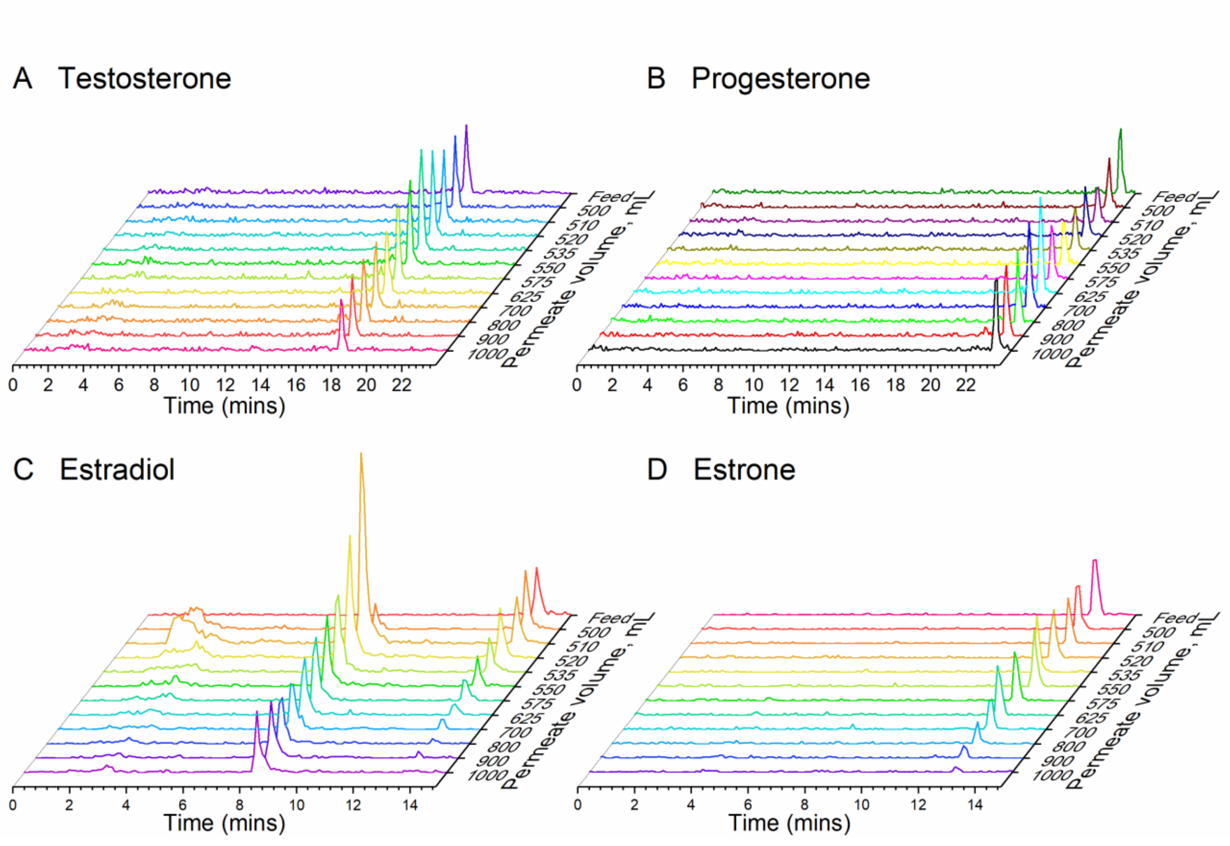

Supplementary Figure 11. UHPLC-FSA chromatograms of E2 during the electrochemical degradation with increasing accumulated permeate volume at varying flux (A) testosterone, (B) progesterone, (C) estradiol and (D) estrone. $c_{f,SH}$ = 1 µg L^{-1}, V_{cell} = 1.6 V, J_f = 150 L m^{-2} h^{-1} (5 mL min^{-1}), 1 mM $NaHCO_3$, 10 mM NaCl, 27.2 mg L^{-1} EtOH, 79.2 mg L^{-1} MeOH, pH 8.2±0.2, 23±1 °C.

No significant byproducts were detected in the UHPLC chromatograms for for T and P.

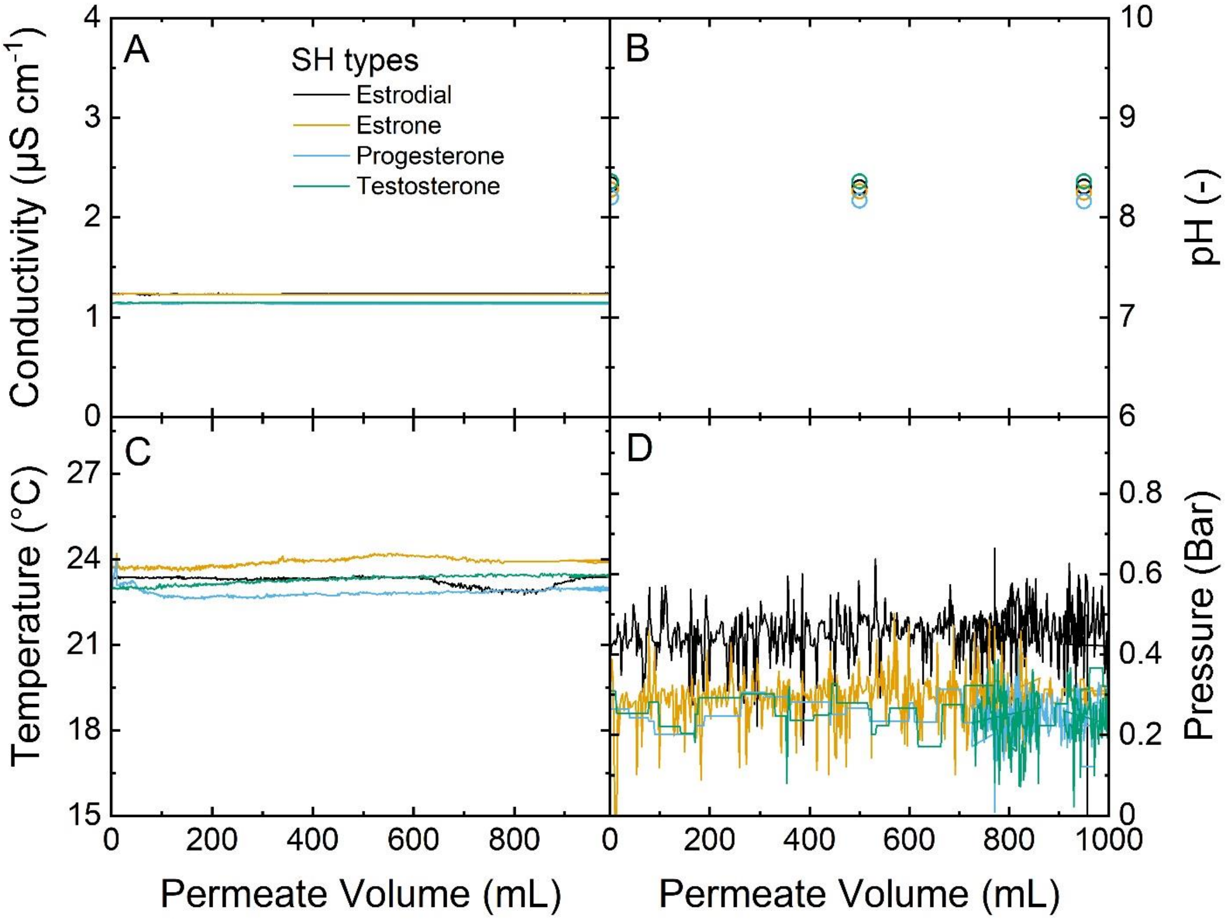

Supplementary Figure 12. Variations of (A) conductivity, (B) pH, (C) temperature conductivity, and (D) transmembrane pressure during electrochemical filtration experiments at varying SH types. $c_{f,SH}$ = 1 μg L^{-1}, V_{cell} = 1.6 V, J_f = 150 L mh (5 mL min^{-1}), 1 mM $NaHCO_3$, 10 mM NaCl, 27.2 mg L^{-1} EtOH, 79.2 mg L^{-1} MeOH, pH 8.2±0.2, 23±1 °C.

The change in the pattern of pressure fluctuations before and after 300 mL permeate volume for P and T was likely due to a decrease in the intervals of data sampling, resulting in a more continuous line before 300 mL.

Supplementary Methods

Supplementary Discussion 3. Experimental system and protocol

Electrochemical filtration system

A custom-built flow-through electrochemical filtration set-up (Figure 1) was used in this work for investigation of SH removal with the CNT electrochemical membrane. The experimental setup is comprehensively equipped with various key components: A thermostatic circulator (Pilot One CC-K6s, Huber, Germany) is connected to a 1-L jacketed glass container to maintain the temperature of the feed solution at 23±1 °C, a peristaltic pump (07528-30, MasterFlex, USA) is utilized to drive the influent solution, a commercial electrochemical filtration cell (CF016A, SterliTech, USA), with its voltage regulated by a direct current power supply (DPPS 60-15, VoltCraft, Germany). The transmembrane pressure is monitored by in-line pressure sensors (0-25 bar, WIKA A-10, Alexander

Wiegand SE & Co. KG, Germany) on both sides of the cell. A pH meter (pH/cond 3320, WTW, Germany) equipped with an electrode (SenTix81, WTW, Germany) and a conductivity sensor (CR-EC, JUMO, Germany) are employed for the measurement of pH and conductivity of the feed solution. Further, the temperature and conductivity of the permeate are assessed using an in-line thermocouple (NI USB-TC01, NI, USA) and another conductivity sensor (CR-EC, JUMO, Germany). Finally, a data acquisition card (DAQ, USB-6000, NI, USA) is integrated into the system for the acquisition and management of data from these various components.

Electrochemical filtration cell

The EMR (Supplementary Figure 13.) used in this work, constructed from durable acrylic, is designed for the study of electrochemical strategies within an electrochemical membrane in a flow-through configuration.

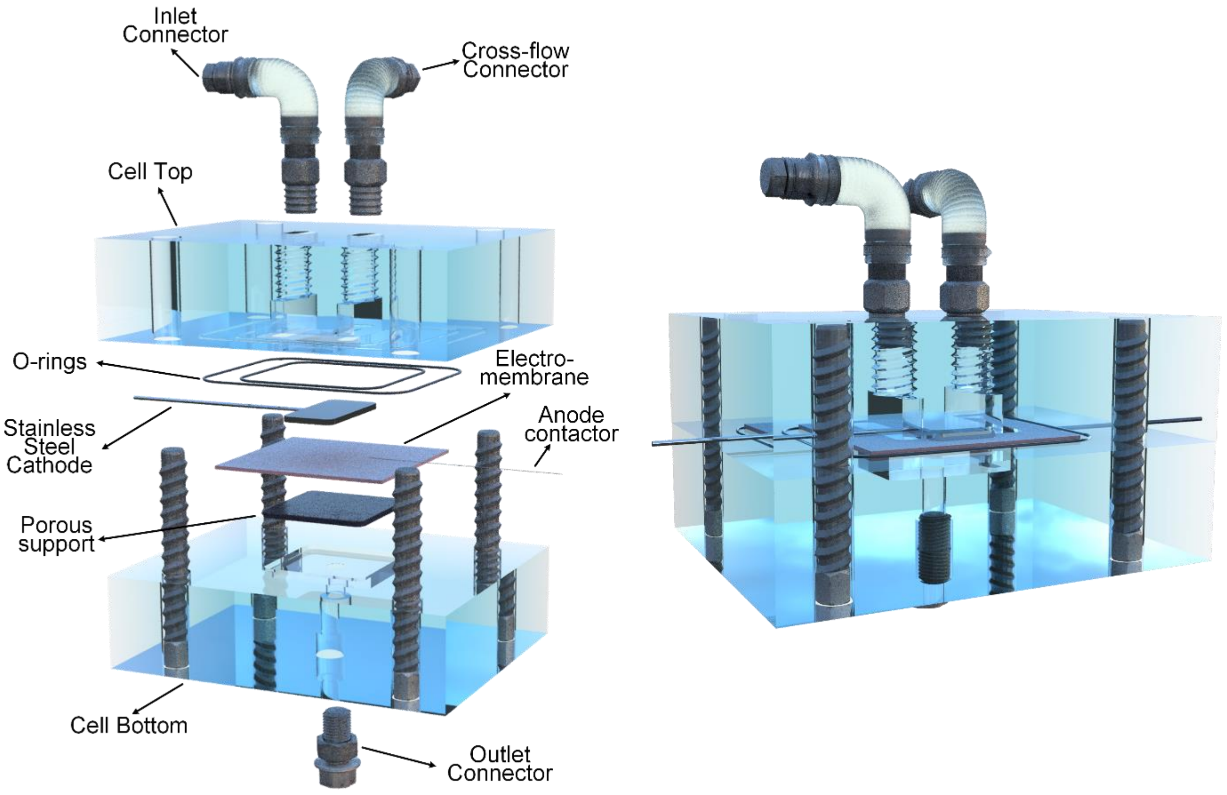

Supplementary Figure 13. Schematic diagram of the electrochemical membrane reactor.

Its central feature is an electrochemical membrane with a working area of 20 cm² (4.5 cm x 4.5 cm), serving as the flow-through anode. Opposite to this, a stainless steel plate positioned 2.3 mm apart from the membrane functions as the cathode, with good conductivity and strength. Other design of the cell includes a titanium rod connected to the cathode and a platinum (Pt) wire atop the membrane, facilitating electrical connections to an external power supply.

Electrochemical filtration protocol

All electrochemical filtration experiments were conducted in dead-end mode, following a protocol outlined in Supplementary Table 1.

Supplementary Table 1. Experiment protocol for electrochemical filtration experiments.

No.	Step	Conditions	Justification
1	Purging of the pump with MilliQ	90 mL min^{-1} for 30 s of MilliQ with valve ① & valve ③ open, and with valve ② closed	-Remove the air bubbles in the pump head
2	Flushing the system with MilliQ	40 mL min^{-1} for 2 min of MilliQ with valve ① closed, and valve ② & ③ open (cross-flow)	Remove the bubbles in the system
3	Permeability	- Close valve ① & ③ closed, and open valve ② (dead-end)<br>- Run different flow rates (10, 7.5, 5, 2, and 1 mL min^{-1}) for 5 min and collect the pressure data	Evaluate the membrane permeability before the experiments
4	Pure water flux	Run 10 mL min^{-1} (300 L m^{-2} h^{-1}) of MilliQ for 40 min and collect the pressure data	- Achieve stable flux prior to the experiment
5	Preparation of conductivity and pH meters	Put the conductivity and pH in the feed tank and turn them on	Monitor the conductivity and pH in the feed solution
6	Power supply connection and setting	- Connect the anode and cathode to the power supply<br>- Set the required applied voltage	Apply electric potential on the membrane
7	Setting pump flowrate	Set the required pump flowrate	Drive the influent at a constant flow rate
8	Preparation of vials for permeate sample	Label the sample vials and put under the switching valve for taking samples during the filtration	
9	Adsorption	- Switch the inlet tube to 1 μg L^{-1} SH feed solution<br>- Run with power supply off for 500 mL<br>- Take a sample of the feed solution before starting adsorption phase	Determine the adsorption of SHs on the EM
10	Electrochemical degradation	-Power supply is switched on while the working flowrate is still kept<br>-The volume of filtered SHs is 500 mL	Determine the degradation efficiency of SHs on the EM
11	Flushing the system with MilliQ	Repeat step 1 and 2 with MilliQ	Remove the residual SH from the system
12	Permeability	Repeat step 3 with MilliQ	Evaluate the membrane permeability after the experiments
13	Lines cleaning	Clean each line of the switching valve using MilliQ at 10 mL min^{-1} for 5 min	Clean the lines for sample collections

Supplementary Discussion 4. Membrane characterization

Pure water flux

Pure water flux of the electrochemical CNT membrane was measured over the transmembrane pressure, as shown in Supplementary Figure 14..

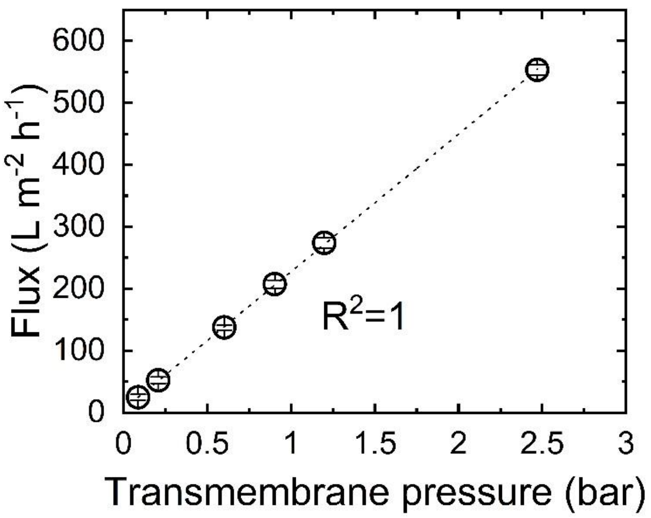

Supplementary Figure 14. Pure water flux for the fresh electrochemical CNT membrane.

The pure water flux of the CNT membrane across the transmembrane pressure ranging from 1 to 20 bar demonstrated a perfect linear relationship (R^2=1). Error bars represent propagated error from operational parameter variations and analytical error.

Permeability of the membrane was determined to be 218±1 L m^{-2} h^{-1}.bar as the slope of the linear fit of water flux vs. transmembrane pressure.

Measurement of membrane surface resistivity

The intrinsic resistivity of the CNT membrane was measured using a four-point probe (4PP, Supplementary Figure 15) [1] with specifics provided in

Supplementary Table 2.

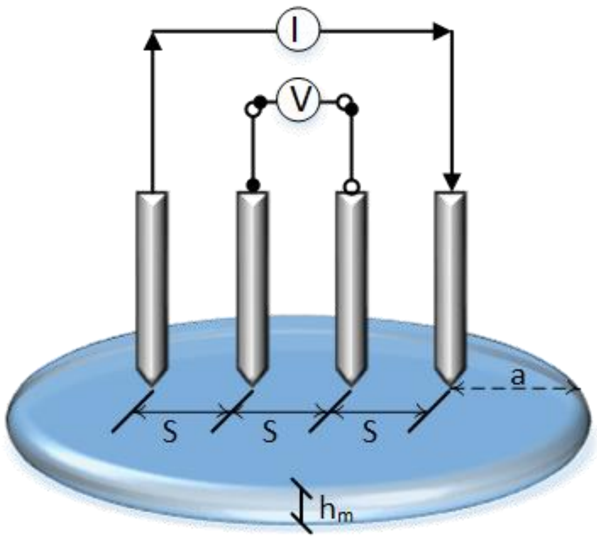

Supplementary Figure 15. Schematic diagram of a collinear four-point probe measurement.

Supplementary Table 2. Specifications of the four-point probe source meter and probe head.

**Parameter**	**Working value**
Limit of current	1.5 A
Limit of voltage	20 V
Spacing between tips, s	1.016 mm
Pressure on each probe tip	180 gram
Tip radius	0.254 mm

In the 4PP measurement, current I passes between the two outer probes while the resultant voltage drop V is measured across the two inner probes. Separating current injection and voltage measurement eliminates the contact resistance because the two probes that detect voltage, draw little current [2]. To avoid the edge effects, which refered to disturbances in the electrical field near the edges of the material, it was ensured that the distance from any probe to the nearest boundary (a) was at least five times greater than the probe spacing (1.016 mm) [3]. The resistivity measured by 4PP is determined to be $7.2{\cdot}10^{-4}$ Ω·m using Eq. (S1):

$$\rho = \frac{V_c}{I} h_m f \qquad \text{(S1)}$$

where I (A) is the applied current, V_c (V) is the detected voltage, h_m is the thickness of the sample ($2{\cdot}10^{-6}$ m [4]), and f is a geometric factor. Since $h_m \leq 5s$, f is determined as $\pi/\ln 2$ [3].

Zeta-potential

To characterize the surface charge of the CNT membrane as a function of pH, the zeta-potential were measured at pH ranging from 2 to 10 in 10 mM NaCl electrolyte solution (Supplementary Figure 16).

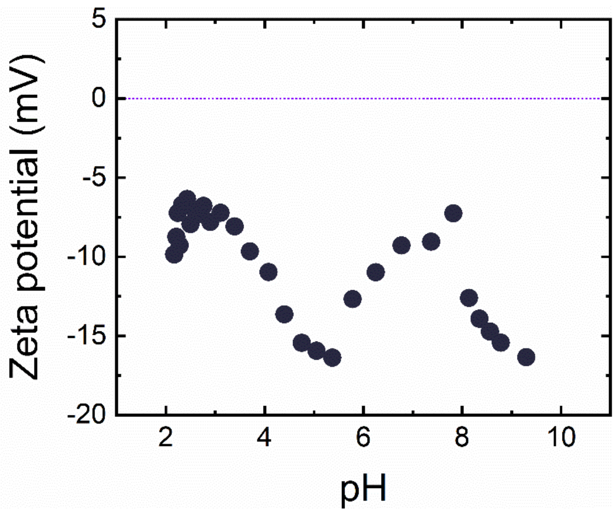

Supplementary Figure 16. Zeta-potential of CNT membranes as a function of pH. 10 mM NaCl electrolyte solution, pH adjustment using 0.05 M HCl and 0.05 M NaOH.

The CNT membrane exhibited a negative surface charge from pH 2 to 10, likely attributable to the –COOH groups on the surface of the CNTs.

Three electrode set-up

The electrochemical surface potential of the CNT membrane were assessed through an open circuit potential measurement in a three-electrode configuration. Supplementary Figure 17 illustrates this setup, comprising a CNT membrane as the working electrode, a platinum (Pt) wire as the counter electrode, and a Ag/AgCl electrode acting as the reference electrode.

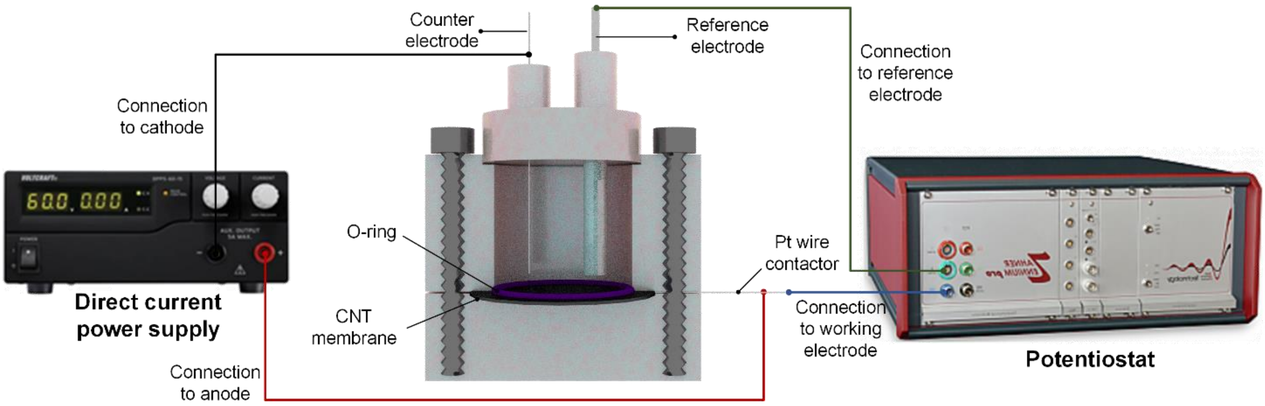

Supplementary Figure 17. Three-electrode set-up for membrane surface potential measurement [5].

Membrane surface potential at varying cell voltage

Supplementary Figure 18. shows the electrochemical surface potential of the CNT membrane measured at varying applied cell voltage from 0.6 to 2.5 V.

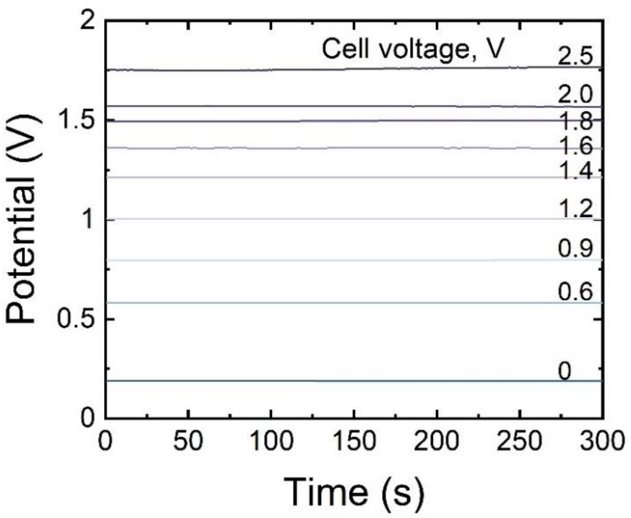

Supplementary Figure 18. Membrane surface potentials *vs*. Ag/AgCl at varying cell voltage in 10 mM NaCl and 1 mM $NaHCO_3$.

The surface potentials of the CNT membrane remain stable over time across all cell voltages. The surface potential without application of a voltage was determined to be 0.19 V in the electrolyte containing 10 mM NaCl and 1 mM $NaHCO_3$. In 10 mM NaCl and 1 mM $NaHCO_3$ electrolyte, the CNT membrane exhited lower surface potentials than the apllied cell voltage (ranged from 0.6 to 2.5 V), primarily due to the Ohmic drop across the electrolyte, electrode materials, and interfaces.

Supplementary Discussion 5. Characterics and analytical methods for radiolabeled steroid hormone

Characteristics of steroid hormone

Four SH types, estrone (E1), β-estradiol (E2), progesterone (P), and testosterone (T), were used as surrogates micropollutants in aquatic environment in this study, of which the structure and properties are summarized in Supplementary Table 3. [6-16].

Supplementary Table 3. Chemical structures and selected properties of E1, E2, P, and T [6-16].

	**Estrone (E1)**	**Estradiol (E2)**	**Progesterone (P)**	**Testosterone (T)**
Chemical structure	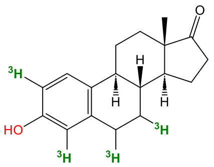	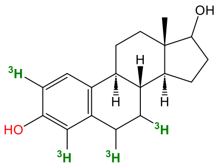	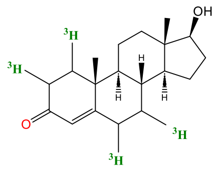	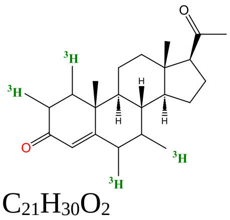
Formula	$C_{18}H_{22}O_2$	$C_{18}H_{24}O_2$	$C_{19}H_{28}O_2$	$C_{21}H_{30}O_2$
Molecular weight (g mol^{-1})	270.4	272.4	314.5	288.4
pKa[1]	10.3-10.8	10.2-10.7	-	-
Stokes diameter (nm)[2]	0.79-0.82	0.80	0.86	0.82
Solubility in H_2O (g L^{-1})[3]	0.80-1.30 (25°C)	0.16-5.00 (25°C)	7.9-16.8 (25°C)	20.0-48.0 (25°C)
Electron-affinity[4]	-0.42	-0.50	0.05	0.12

[1]Data are adapted from literatures [6-8]. [2]Data are adapted from literatures [9]. [3]Data are adapted from literatures [10-15]. [4]Electron-affinity is a measure of the tendency of a molecule to attract electrons. a compound with high electron affinity is more likely to attract electrons, whereas a compound with low electron affinity would be less likely to attract electrons and thus could be more susceptible to reactions with nucleophiles rather than electrophiles [17-19]. The data are adapted from previous work [16].

Elution parameters for UHPLC

The UHPLC-FSA analytical method utilized in this study for analyzing SH concentrations was adapted from Lyubimenko *et al.* [20], with a modification in the elution flow rate from 0.25 to 0.2 mL min^{-1}, and injection volume from 100 to 200 mL to reduce the operating pressure using the same instrument. The detailed elution parameters are presented in Supplementary Table 4.

Supplementary Table 4. Elution parameters for the UHPLC-FSA analytic procedure.

**Parameters**	**Conditions**
Column	150 mm x 2.1 mm, 1.7 µm C18, 100 Å
UHPLC flow rate, mL min^{-1}	0.2
Mobile phase	MeOH/Water (40/60)
Mobile phase gradient time, min	15
Elute gradient	Initial MeOH of 40% increase linearly to 80%
Injection volume, µL	200
Column temperature, °C	50
Autosampler temperature, °C	4

Calibration curves for UHPLC and LSC

Supplementary Figure 19. presents the calibration curve for E2, performed using a range of E2 concentrations (0.1 – 100 ng L^{-1}) for UHPLC and LSC analyses.

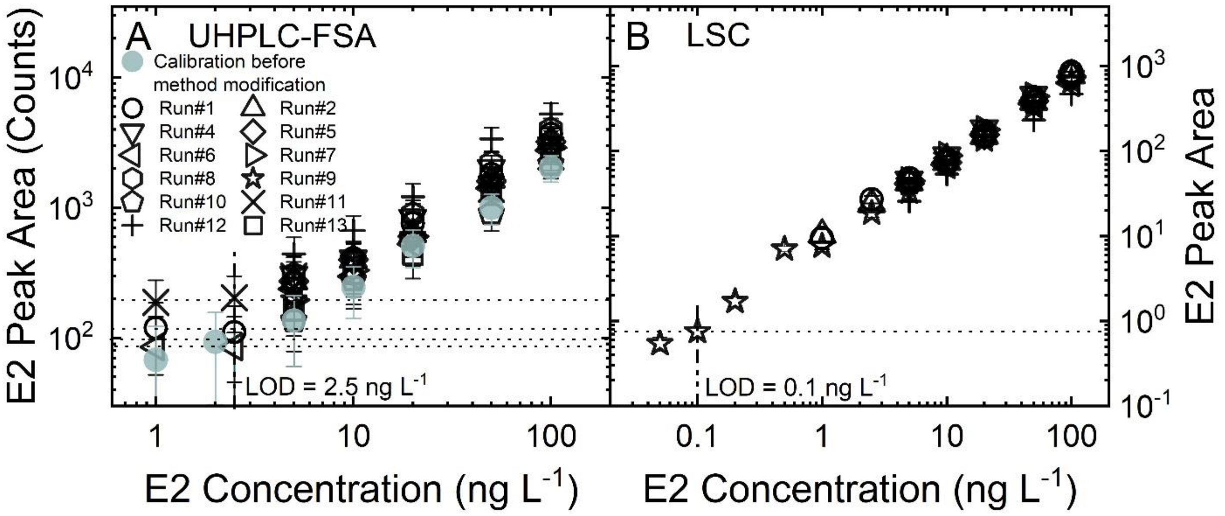

Supplementary Figure 19. E2 calibration curve for (A) UHPLC-FSA and (B) LSC in log-scale. The data for calibration before method modification was adapted from [21]. Error bars represent propagated error from operational parameter variations and analytical error.

The UHPLC-FSA demonstrated good linear relationships across the E2 concentration ranging from 2.5 to 100 ng L^{-1}. The UHPLC-FSA achieved a LOD at 2.5 ng L^{-1}, maintaining comparability with

results prior to the modification of operational parameters. The LSC demonstrated strong linear correlations for E2 concentrations spanning from 0.1 to 100 ng L^{-1} and a LOD of 0.1 ng L^{-1}.

Supplementary Discussion 6. Data processing

Membrane performance

Table S5 shows the calculation of electric conductivity (σ, S m^{-1}), permeate flow rate (Q_p, L h^{-1}), water flux (J_w, L m^{-2} h^{-1}), permeability (L, L m^{-2} h^{-1} bar), and mean hydraulic residence time (t_r, h) of the CNT layer on the electrochemical membrane.

Supplementary Table 5. Calculation parameter and equations.

Variable	Equation	Unit	
Electric conductivity (σ)	$\sigma = \frac{ln2I}{\pi V_c h_m}$	S m^{-1}	(S2)
Permeate flow rate (Q_p)	$Q_p = \frac{V_{t2} - V_{t1}}{t2 - t1} = \frac{1000 \cdot (m_{t2} - m_{t1})}{(t2 - t1) \cdot \rho}$	L h^{-1}	(S3)
Water flux (J_w)	$J_w = \frac{Q_p}{A}$	L m^{-2} h^{-1}	(S4)
Permeability (L)	$L = \frac{J_w}{\Delta P}$	L m^{-2} h^{-1} bar	(S5)
Mean residence time (t_r)	$t_r = \frac{V_p}{Q_f} = \frac{h_m \varepsilon}{10^{-3} \cdot J_w}$	h	(S6)

where I (A) is the applied current, V_c (V) is the detected cell voltage, h_m is the thickness of the CNTs layer ($2 \cdot 10^{-6}$ m [4, 22]). Measurement of a standard indium tin oxide (ITO) film with known sheet resistance of 15 Ω $square^{-1}$ showed the error of electric conductivity was within ± 10%.

V_{t2} and V_{t1} (L) were the accumulated volume of the permeate concerted from the permeate mass at time $t2$ and $t1$ (h). The density of pure water (ρ) at the operating temperature of 23 °C (997.3 kg m^{-3} [23]), was utilized to convert the mass of the permeate (m_{t2} and m_{t1}, kg) at $t2$ and $t1$ into its corresponding volume for all experiments.

A is the effective membrane surface area ($2 \cdot 10^{-2}$ m^2), ΔP is the transmembrane pressure (bar), Q_f is the feed flow rate (L h^{-1}).

ε is the porosity of the CNT layer on the membrane support, which is estimated to be 66% from the porosity of a similar CNTs membrane [24].

Determination of SHs removal

To reduce potential errors in calculating R, arising from variations in data points, the experimental data of the normalized SH concentration in permeate ($c_{p,SH}/c_{f,SH}$) *vs.* the accumulated permeate

volume (V_p) were analysed through a fitting procedure [25]. This process utilized a power model ($y = a \cdot x^b$) within the V_p range between 520 and 1000 mL. Subsequently, the $c_{p,eq}/c_f$ was obtained from the curve fitted to the data at V_p = 1000 mL, as depicted in Supplementary Figure 20..

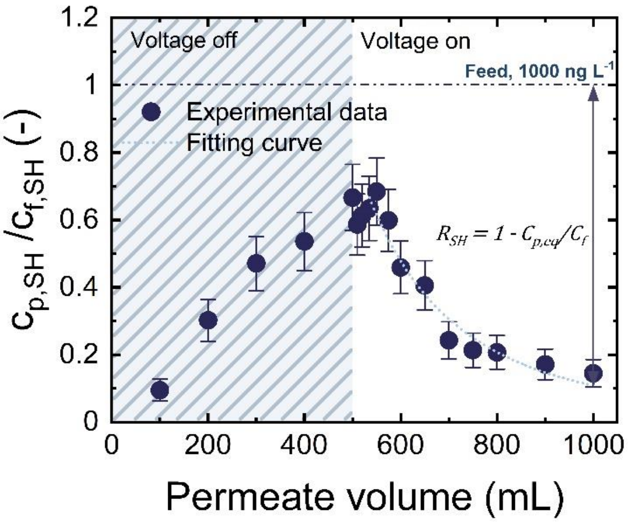

Supplementary Figure 20. Example of determination of SH removal (R) by fitting the $c_{p,SH}/c_f$ data in the range of 550 – 1000 mL of permeate volume. Error bars represent propagated error from operational parameter variations and analytical error.

Determination of the total mass of removed SH with the membrane

To calculate the total mass of SH removed with the EMR over the entire experiment including adsorption and degradation phase, the dataset of c_p against V_p was analyzed using a polynomial fitting model ($y = A + B \cdot x + C \cdot x^2 + D \cdot x^3$) for the adsorption phase and a power model ($y = a \cdot x^b$) for the degradation phase. The integration of the fitting curve with permeate volume as the independent variable, provided the cumulative mass of SH in permeate and the total removal mass of SH can be subsequently quantified. An illustrative example of this quantification process for the overall mass of removed E2 in the EMR is presented in Supplementary Figure 21..

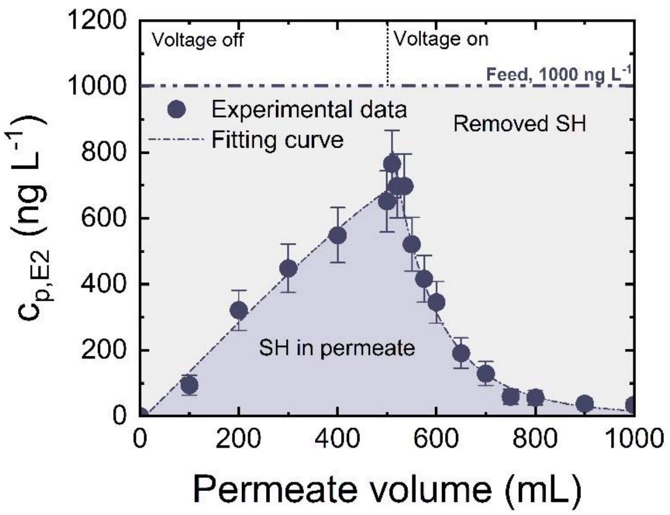

Supplementary Figure 21. Example of determination of total mass of E2 removed within the EMR. Error bars represent propagated error from operational parameter variations and analytical error.

In Supplementary Figure 21., the purple-shaded area indicates the accumulative mass of E2 in the permeate, while the total mass of E2 removed within the EMR during the 1 L filtration experiment is represented by the areas shaded in grey.

Determination of the total mass of byproducts

Figure S7 illustrates the quantification of the total mass of byproducts in the permeate. This was achieved by fitting the experimental data obtained from UHPLC-FSA and LSC and then integrating the resulting curve.

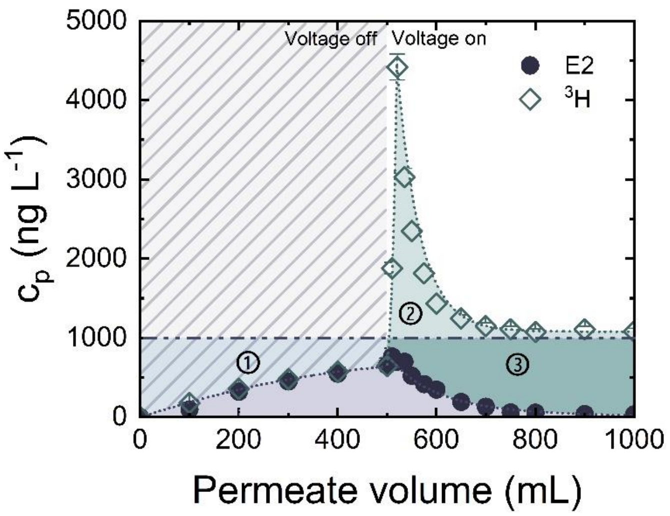

Supplementary Figure 22. Example of determination of total byproducts formed and desorbed from the membrane within the EMR. Error bars represent propagated error from operational parameter variations and analytical error.

The area shaded in green represents the cumulative mass of byproducts that formed and were desorbed from the membrane throughout the electrochemical degradation process.

Determination of the mass of electrochemical adsorbed and degraded SH

To determine the total mass of SH adsorbed on the membrane (under the assumption that all byproducts are fully desorbed), the experimental data of the total ^{3}H activity, as measured by LSC, are integrated over the range of permeate volumes (Supplementary Figure 23). The total mass of degraded SH is then calculated by subtracting the adsorbed SH from the removed SH.

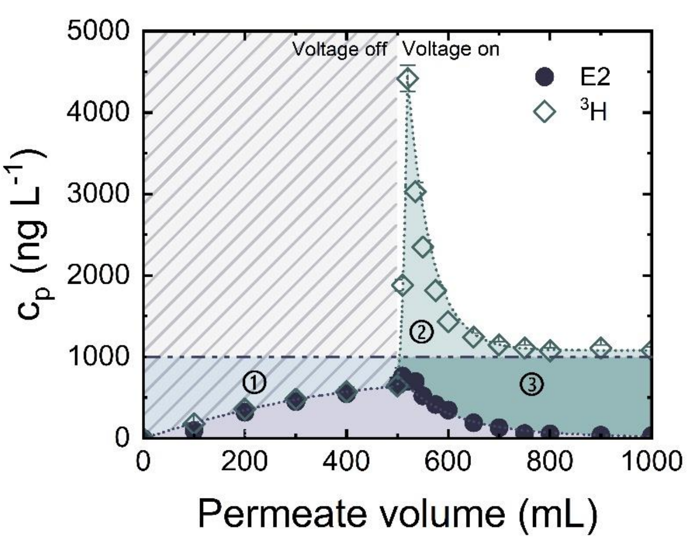

Supplementary Figure 23. Example of determination of the contribution of electrochemical adsorption and degradation. Error bars represent propagated error from operational parameter variations and analytical error.

Supplementary Figure 23 illustrates the calculation of the total mass of adsorbed SH, determined as the difference between areas ① and ②. Consequently, the mass of degraded SH is quantified by subtracting the adsorbed SH (① - ②) from the total removed SH (① + ③). It was observed that the total mass of degraded SH is equivalent to the mass of the formed byproducts (② + ③), which arises from the assumption that all degradation byproducts are completely desorbed from the membrane.

Mass balance analysis for the pre-adsorption experiments

As an illustration of the mass balance analysis conducted using the integrated UHPLC-FSA and LSC method, the contribution of degraded, adsorbed, and unremoved E2 in the experiments with and without pre-adsorption, is shown in Supplementary Figure 24.

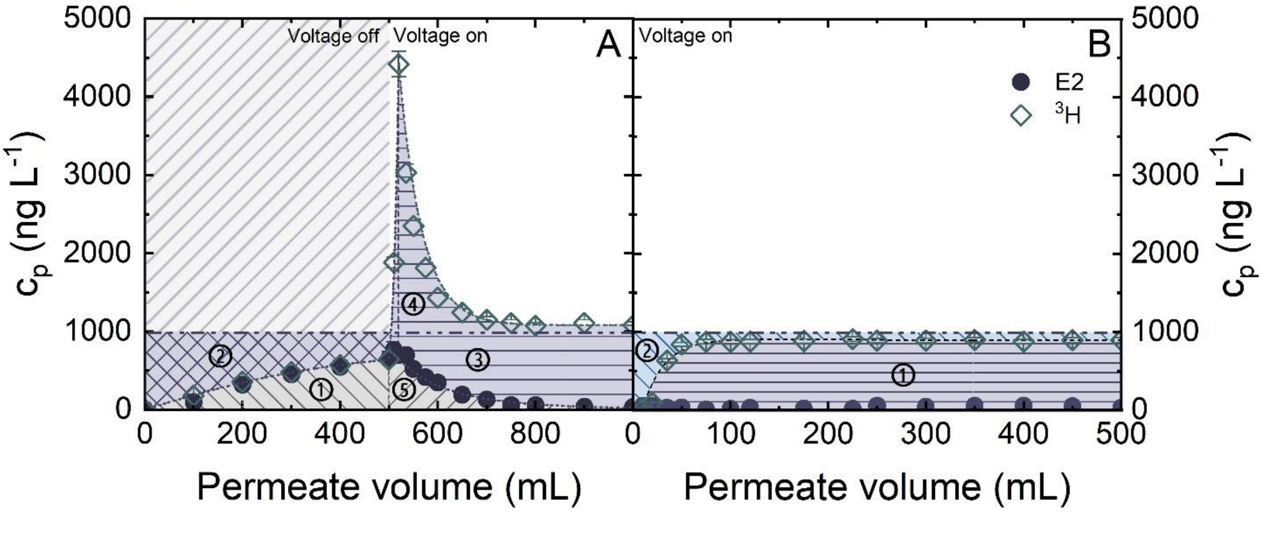

Supplementary Figure 24. Illustration of the mass balance analysis of the degraded, adsorbed, and unremoved E2 in the experiments with and without pre-adsorption. Error bars represent propagated error from operational parameter variations and analytical error.

In the pre-adsorption experiment (Supplementary Figure 24A), prior to activating the voltage, 19±4% of the initial E2 mass was released into the permeate (unremoved), corresponding to the integrated area ① over the volume range of 0-500 mL. Additionally, 31±4% was adsorbed onto the membrane (area ②). After activating the voltage, 5±2% of the E2 passed through the system unremoved, as determined by the integrated area ⑤ for the 500-1000 mL permeate volume. In total, 61±7% of E2 was degraded (area ③+④) during the degradation phase (500-1000 mL), while 15±3% remained adsorbed on the membrane (area ②-④).

In the experiment without pre-adsorption (Supplementary Figure 24B), 77±3% of E2 was degraded (area ①) and 22±4% (area ②) was adsorbed onto the membrane, while the remaining 2±2% was released into the permeate.

Supplementary Discussion 7. Error estimation

In this study, an error propagation method was employed to estimate experimental error, as described previously [26]. The following sections provide the detailed method of the error determination considering different variabilities, including: i) preparation of feed solutions (Δprep) due to the uncertainties of pipetting and volumetric variations; ii) EMR system caused by the uncertainties of its components (ΔS); and the quantification of SH concentration and ^3H activity using the analytical tools (Δanal) - UHPLC-FSA (ΔUHPLC) and LSC (ΔLSC). Supplementary Table 6. summarized the sources and their estimated relative errors that contribute to the total the error in research data.

Supplementary Table 6. Error sources and their estimated relative errors in the SH concentration anaylysis.

**Parameter**	**Error source**	**Justification**	**Calculation**	**Relative error (%)**
Sample preparation (Δprep)	Pipettes (Δpip); Volumetric flasks ($\Delta vol.flasks$)	The feed concentration (c_p) is variable with the volume of SH stock and MilliQ measured by pipettes and flasks	$\Delta \mathrm{Prep} = \sqrt{\Delta pip^2 + \Delta vol.flasks^2}$	4
Experimental system (ΔS)	Flow rate (ΔQ); Electrolyte conductivity (Δσ) Cell voltage ($\Delta voltage$)	Flow rate, electrolyte conductivity, and cell voltage affects the SH removal by varying the number of SH molecule to be treated per unit of time, electrolyte resistance, and anodic potential	$\Delta S = \sqrt{\Delta Q^2 + \Delta\sigma^2 + \Delta voltage^2}$	6

UHPLC system (ΔUHPLC)	SH concentration in the sample is variable with the resolution of UHPLC		1.1
FSA detector (ΔFSA)	^{3}H activity in the sample is variable with the error of analysis detector	$\Delta\text{FSA} = 2 \cdot \frac{\sigma_{NC}}{N_{NC}}$ $\sigma_{NC} = \sqrt{{\sigma_{TC}}^2 + {\sigma_{BG}}^2}$	10-16
LSC system (ΔLSC)		$\Delta C_{LSC} = 10.536 \cdot {c_{cLSC\ measured}}^{-0.5441}$	2-6

where: σ_{NC} - standard deviation of net counts, N_{NC} - net number of counts, σ_{TC} - standard deviation of number of total counts, σ_{BG} - standard deviation of background counts, $c_{cLSC\ measured}$ is the permeate concentration measured by LSC

The relative error for each system parameter was determined with the data acquired by different sensors using the following equations:

$$\Delta y_{abs} = \frac{y_{max} - y_{min}}{2} \quad \text{(S7)}$$

$$\Delta y_{rel} = \frac{\Delta y_{abs}}{\bar{y}} \quad \text{(S8)}$$

where Δy_{abs} is the absolute error of the system parameter y, y_{max} and y_{min} are the maximum and minimum values of y, respectively for sufficient repeats, Δy_{rel} is the relative error, and $\bar{y}$ is the mean value. The approaches described in previous work were employed for the error propagation of analytic tools, UHPLC-FSA [20] and LSC [27].

Error analysis of SH concentration, ^{3}H activity, normalized concentration and removal

The total relative error of the SH concentration and ^{3}H activity (ΔE) quantified through HPLC-FSA and LSC was estimated using Eq. (S9).

$$\Delta\text{E} = \sqrt{\Delta\text{Prep}^2 + \Delta S^2 + \Delta\text{anal}^2} \quad \text{(S9)}$$

To make this calculation clear, as an example, the errors for feed concentrations measured using UHPLC-FSA and LSC in variation of sample preparation (4%) and analytical method (1.1% for UHPLC, 10-16% for FSA, and 2-6% for LSC) are reported (Supplementary Figure 25.). The errors of feed concentrations analyzed with UHPLC-FSA were determined to be ±11-17%, and those measured through LSC were estimated to be ±4-7% using Eq (S9).

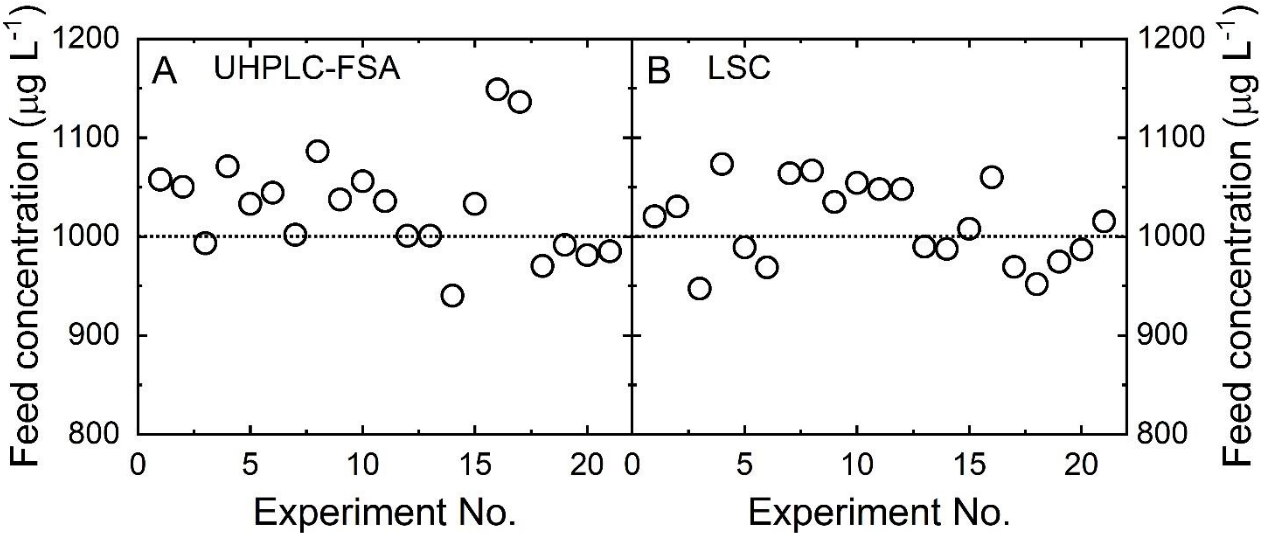

Supplementary Figure 25. Feed concentrations measured using UHPLC-FSA and LSC for the electrochemical filtration experiments.

Supplementary Figure 25. showed that the feed concentrations measured by UHPLC-FSA ranged from 940 to 1149 μg L^{-1}, while those measured by LSC varied between 947 and 1073 μg L^{-1}. The errors associated with these measurements fall within the range calculated using the error propagation method.

The relative error of normalized SH concentration and ^{3}H activity in the permeate ($\Delta c_p/c_f$) is directly related to the error of c_p and c_f, which is determined using the equation:

$$\Delta(c_p/c_f) = \frac{c_p}{c_f} \cdot \sqrt{\Delta c_f{}^2 + \Delta c_p{}^2} = \frac{c_p}{c_f} \cdot \sqrt{(\Delta \mathrm{Prep}^2 + \Delta \mathrm{anal}^2) + \Delta E^2} \tag{S10}$$

Correspondingly, the relative error of removal ($R = 100 \cdot (1 - c_p/c_f)$) is calculated *via* the equation:

$$\Delta R = \frac{c_p}{c_f} \cdot \Delta(c_p/c_f) \tag{S11}$$

Error analysis of total mass of removed SH and rate of SH removal

According to the calculation of total mass removal either by adsorption or degradation (Eq. (S12)), error of the total mass removal (Δm_{rem}) is determined by the error of c_f and the accumulative SH mass in the permeate (m_p). The absolute error of m_p ($\Delta m_{p,abs}$) was calculated by fitting the data of $c_p + \Delta c_{p,abs}$ and $c_p - \Delta c_{p,abs}$, and then the $\Delta m_{p,abs}$ can be determined by integrating the fitting curves (Supplementary Figure 26.).

$$m_{rem} = m_f - \sum_{i=1}^{n} m_{p,i} \tag{S12}$$

where $V_{p,i}$ and $c_{p,i}$ are the volume and concentration of the permeate sample i, n is the total number of the permeate samples.

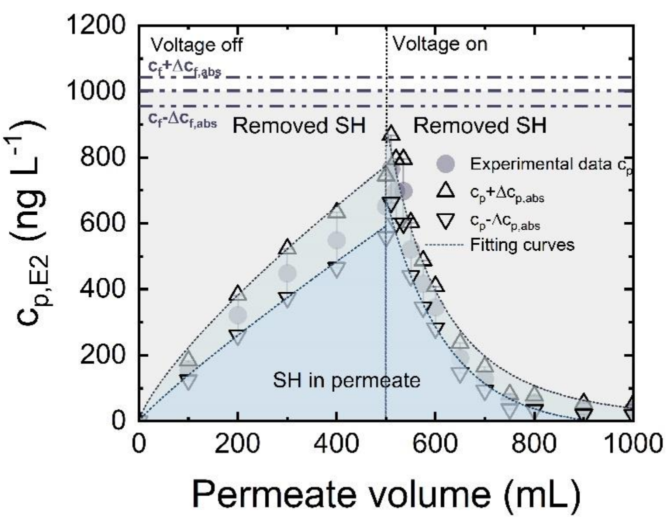

Supplementary Figure 26. Example of determination of the absolute error of total SH mass in the permeate. Error bars represent propagated error from operational parameter variations and analytical error.

The $\Delta m_{p,abs}$ was determined by half the difference in the integrated area under the fitting curve for $c_p + \Delta c_{p,abs}$ and $c_p - \Delta c_{p,abs}$, presented by half the green area in Supplementary Figure 26.. Thus, the absolute error for the removed SH ($\Delta m_{rem,abs}$) can be calculated using the equation below.

$$\Delta m_{rem,abs} = \frac{m_{rem,max} - m_{rem,min}}{2} = \frac{\{(c_f + \Delta c_{f,abs}) - (c_p - \Delta c_{p,abs})\} - \{(c_f - \Delta c_{f,abs}) - (c_p + \Delta c_{p,abs})\}}{2} \quad \text{(S13)}$$

According to the definition of the apparent rate of SH removal ($r'_{rem} = \frac{m_{rem}}{t \cdot M \cdot A}$), the relative error of r'_{rem} ($\Delta r'_{rem}$) was directly determined by the Δm_{rem}, which can be calculated using:

$$\Delta r'_{rem} = \Delta m_{rem} = \frac{\Delta m_{rem,abs}}{m_{rem}} \quad \text{(S14)}$$

Supplementary References

1. PAUW L.J.v.d., A method of measuring specific resistivity and Hall effect of discs of arbitrary shape, *Philips Research Reports* **13**, 1-9 (1958).
2. Kim J.-K., Zhang Y., Lee D.-W., A smart microfour-point probe with ultrasharp in-plane tips, *Review of Scientific Instruments* **80**, 045107 (2009).
3. Lu Y., Santino L.M., Acharya S., Anandarajah H., D’Arcy J.M., Studying electrical conductivity using a 3D printed four-point probe station, *Journal of Chemical Education* **94**, 950-955 (2017).
4. Zhu X., Dudchenko A.V., Khor C.M., He X., Ramon G.Z., Jassby D., Field-induced redistribution of surfactants at the oil/water interface reduces membrane fouling on electrically conducting carbon nanotube UF membranes, *Environmental Science & Technology* **52**, 11591-11600 (2018).
5. Plate material evaluating cell, Teflon. ALS, Japan. https://www.als-japan.com/1127.html. Access date: 19/07/2024.
6. Perrin D.D., Dempsey B., Serjeant E.P., *pKa prediction for organic acids and bases*, Chapman and Hall, London ; New York, (1981).
7. Lewis K.M., Archer R.D., pKa values of estrone, 17β-estradiol and 2-methoxyestrone, *Steroids* **34**, 485-499 (1979).
8. Bhandari A., Surampalli R.Y., Adams C.D., Champagne P., Ong S.K., Tyagi R.D., Zhang T.C., *Contaminants of emerging environmental concern*, American Society of Civil Engineers, Reston, Virginia, USA, (2009).
9. Worch E., Eine neue gleichung zur berechnung von diffusionskoeffizienten gelöster stoffe, *Vom Wasser* **81**, 289-297 (1993).

10. Shareef A., Angove M.J., Wells J.D., Johnson B.B., Aqueous solubilities of estrone, 17β-estradiol, 17α-ethynylestradiol, and bisphenol A, *Journal of Chemical & Engineering Data* **51**, 879-881 (2006).
11. Bischoff F., Pilhorn H.R., The state and distribution of steroid hormones in bilogic system: III. Solubilities of testosterone, progesterone, and α-estradiol in aqueous salt and protein solution and in serum, *Journal of Biological Chemistry* **174**, 663-682 (1948).
12. Kabasakalian P., Britt E., Yudis M.D., Solubility or some steroids in water, *Journal of Pharmaceutical Sciences* **55**, 642 (1966).
13. Haskins A.L., Solubility of progesterone in water and in saline, *Proceedings of the Society for Experimental Biology and Medicine* **70**, 228-229 (1949).
14. Lange W.E., Amundson M.E., Soluble steroids I: Sugar derivatives, *Journal of Pharmaceutical Sciences* **51**, 1102-1106 (1962).
15. Lata G.F., Dac L.K., Steroid solubility studies with aqueous solutions of urea and ureides, *Archives of Biochemistry and Biophysics* **109**, 434-441 (1965).
16. Sudhakaran S., Calvin J., Amy G.L., QSAR models for the removal of organic micropollutants in four different river water matrices, *Chemosphere* **87**, 144-150 (2012).
17. Liu G., Zhang T., Wang T., Yamashita H., Zhao Y., Qian X., Peroxydisulfate activation by photo-generated charges on mesoporous carbon nitride for removal of chlorophenols, *Applied Catalysis B: Environmental* **296**, 120370 (2021).
18. Foote C.S., Peters J.W., Chemistry of singlet oxygen. XIV. Reactive intermediate in sulfide photooxidation, *Journal of the American Chemical Society* **93**, 3795-3796 (1971).
19. DeRosa M.C., Crutchley R.J., Photosensitized singlet oxygen and its applications, *Coordination Chemistry Reviews* **233-234**, 351-371 (2002).
20. Lyubimenko R., Richards B.S., Turshatov A., Schäfer A.I., Separation and degradation detection of nanogram-per-litre concentrations of radiolabelled steroid hormones using combined liquid chromatography and flow scintillation analysis, *Scientific Reports* **10**, 7095 (2020).
21. Liu S., Edara P.C., Schäfer A.I., Influence of organic matter on the photocatalytic degradation of steroid hormones by TiO_2-coated polyethersulfone microfiltration membrane, *Water Research* **245**, 120438 (2023).
22. Duan W., Ronen A., Walker S., Jassby D., Polyaniline-coated carbon nanotube ultrafiltration membranes: Enhanced anodic stability for in situ cleaning and electro-oxidation processes, *ACS Applied Materials & Interfaces* **8**, 22574-22584 (2016).
23. Kell G.S., Density, thermal expansivity, and compressibility of liquid water from 0. deg. to 150. deg.. Correlations and tables for atmospheric pressure and saturation reviewed and expressed on 1968 temperature scale, *Journal of Chemical and Engineering data* **20**, 97-105 (1975).
24. Dudchenko A.V., Rolf J., Russell K., Duan W., Jassby D., Organic fouling inhibition on electrically conducting carbon nanotube–polyvinyl alcohol composite ultrafiltration membranes, *Journal of Membrane Science* **468**, 1-10 (2014).
25. Lotfi S., Fischer K., Schulze A., Schäfer A.I., Photocatalytic degradation of steroid hormone micropollutants by TiO_2-coated polyethersulfone membranes in a continuous flow-through process, *Nature Nanotechnology* **17**, 417-423 (2022).
26. Imbrogno A., Nguyen M.N., Schäfer A.I., Tutorial review of error evaluation in experimental water research at the example of membrane filtration, *Chemosphere*, 141833 (2024).
27. Allouzi M., Imbrogno A., Schäfer A.I., Energy barriers for steroid hormone transport in nanofiltration, *Environmental Science & Technology*, (2022).
